# Traumatic Brain Magnetic Resonance Imaging Feature Extraction Based on Variable Model Algorithm in Stroke Examination

**DOI:** 10.1155/2022/4524958

**Published:** 2022-05-30

**Authors:** Zhenghong Wu, Dongqiu Wu, Weiwei Yang, Bing Wan, Sibin Liu

**Affiliations:** ^1^Department of Radiology, Jingzhou Central Hospital, Jingzhou 434020, Hubei, China; ^2^Department of Function, Jingzhou Chest Hospital, Jingzhou 434020, Hubei, China; ^3^Department of Pulmonary and Critical Care Medicine, Jingzhou Chest Hospital, Jingzhou 434020, Hubei, China

## Abstract

The purpose of this study was to explore the diagnostic value of different sequence scanning of nonparametric variable model-based cranial magnetic resonance imaging (MRI) for ischemic stroke. A histogram analysis-based nonparametric variable model was proposed first, which was compared with the parametric deformation (PD) model and geometric deformation (GD) model. Then, 116 patients with acute ischemic stroke were selected as the research subjects. Routine MRI (T2WI, T1WI, FLAIR, DWI, SWI, and 3D TOF MRA) and MR SCALE-PWI were performed. The results showed that the nonparametric variable model algorithm was relatively complete in the actual segmentation results of MRI images, and the display clarity of lesions was better than PD and GD algorithms. The diagnostic sensitivity, specificity, and overall performance of the variable model algorithm were significantly higher than those of the other two algorithms (*P* < 0.05). According to ROC curve analysis, the AUC areas of DWI, SWI, 3D TOF MRA, and MR SCALE-PWI for the diagnosis of ischemic penumbra were 0.793, 0.825, 0.871, and 0.933, respectively. In summary, the segmentation results of MRI images by the nonparametric variable model based on histogram analysis were relatively complete, and the clarity of lesions was better than that of the traditional model. MRI images can effectively identify the occurrence of ischemic stroke. Moreover, MR SCALE-PWI had a good early identification effect on ischemic penumbra, which can reduce unnecessary treatment for patients.

## 1. Introduction

Stroke is the most common neurological disease that threatens human health and life in modern society [1]. Stroke refers to an acute cerebrovascular disease caused by a sudden rupture or obstruction of cerebral vessels that leads to cerebral blood circulation disorder, thereby causing brain tissue damage, which can be divided into ischemic stroke or hemorrhagic stroke [2, 3]. There are many causes of stroke, such as atherosclerosis, atrial fibrillation, heart valve disease, neck or brain tumors, and congenital vascular malformation. Hypertension, hyperlipidemia, diabetes, and bad habits are risk factors for stroke [4, 5]. The disease has the characteristics of high mortality, high incidence, high recurrence rate, high disability rate, and high prevalence rate, which has become the second leading cause of death in the world after ischemic heart disease. Therefore, it is necessary to diagnose and treat stroke early in clinic [6].

Medical imaging technology can present the internal structure of the human body in a noninvasive manner. Researchers and physicians can obtain potential information to save lives through it [7]. With the development of imaging technology these years, the use of imaging means to check stroke has been widely used [8–10]. With the improvement of imaging technology, doctors widely use computed tomography (CT) and magnetic resonance imaging (MRI) to show lesions. However, early brain CT examinations of stroke patients are mostly normal, and low-density lesions usually appear after 24–48 hours, which increases the difficulty of diagnosis and treatment [11]. MRI, an examination that uses strong magnets, radio waves, and a computer to generate detailed pictures of the human body, can accurately display early ischemic infarcts and has a high detection rate in the examination of cerebellar and brainstem infarcts [12, 13]. However, the original MRI images often have problems such as blurred tissue boundaries, spatial aliasing, and partial volume effect, which need to be enhanced by image segmentation technology [14]. The nonparametric deformation model can adapt to the variability of the anatomical structure over time and different individuals, so it can segment, match, and track anatomical structure targets and can consider the constraints from the image and the constraints on the location, size, and shape of the anatomical structure. Therefore, this study intends to optimize the skull MRI images of stroke patients with nonparametric variable model.

In summary, it is necessary to improve the quality of skull MRI images by using various image segmentation algorithms, but its effect still needs to be further improved. Therefore, a histogram analysis-based Gaussian mixture model was adopted in this research to fit the histogram, and a nonparametric variable model based on histogram analysis was proposed. 116 patients with acute ischemic stroke were selected as the research subjects for multisequence MRI scanning. By analyzing the diagnostic performance of MRI multimodal images for ischemic stroke, the evaluation value of different sequence scanning of skull MRI images based on a nonparametric variable model for ischemic stroke was discussed.

## 2. Materials and Methods

### 2.1. Research Objects

One hundred and sixteen patients with acute ischemic stroke admitted to the hospital from February 20, 2019, to July 10, 2021, were selected as subjects aged 25–71 years. The average age was 44.87 ± 2.6 years, including 73 males and 43 females. This study was approved by the ethics committee of the hospital and the patients and their families understood the study and signed informed consent.

Inclusion criteria were as follows: (i) patients with the first onset; (ii) patients without claustrophobia; (iii) patients with focal neurological deficits; (iv) patients only receiving medication; and (v) patients with good compliance.

Exclusion criteria were as follows: (i) patients with mental diseases; (ii) patients with poor image quality; (iii) patients with incomplete clinical data; and (iv) patients who quit the experiment halfway.

### 2.2. MRI Examination

Patients were examined by a 1.5 T superconducting magnetic resonance system with an 8-channel head coil. During the examination, the patient was in the supine position, wearing a matching headphone, and then, the sponge pad was placed between the subject's head and the MRI coil.

Conventional MRI examination included fast spin-echo sequence (T2WI), gradient-echo sequence (T1WI), fast spin-echo sequence (FLAIR), plane echo sequence (DWI), 3D gradient echo sequence (SWI), and 3D time of flight, magnetic resonance angiography (3D TOF MRA). The scanning parameters of each sequence are shown in Table 1.

MR SCALE-PWI scan: after routine scans, gadolinium injection of a glumine contrast agent (0.15 mmol/kg at 3.5 mL/s) was automatically injected with a high-pressure syringe 45 seconds later, followed by injection of 20 mL of normal saline at the same rate. The scan parameters are shown in Table 2. The generated qCBF and qCBV images were transferred to the workstation for processing; the intelligent segmentation model was used to delineate the region of interest (ROI) of lesions, and the size and quantitative values of the ROI were calculated.

### 2.3. Nonparametric Variable Model Based on Histogram Analysis

Based on the histogram analysis of the images, the Gaussian mixture model was employed to fit the histogram, and the obtained image's statistical characteristic parameters were taken as the constraint conditions to replace the stop term regarding image gradient information in the traditional method, thus guiding and controlling the evolution of the curve and completing the image segmentation.

**Figure 1:**
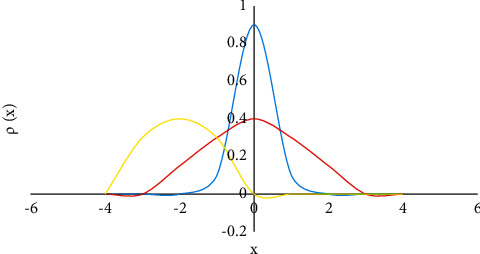
Gaussian mixture model (three Gaussian components).

Gaussian mixture model (GMM) is a probabilistic clustering method [15], which belongs to the generative model. It assumes that all data samples are generated by multivariate Gaussian distribution of a given parameter (Figure 1). Its definition can be expressed as follows:

(1) $$\begin{array}{c} \rho \left(x\right)=\sum_{l=1}^{L}\pi_{l}R\left(\frac{x}{\mu_{l}}\sum_{l}\right). \end{array}$$

*R*(*x*/*μ*_*l*_, ∑_*l*_) is the Gaussian distribution, *μ*_*l*_ represents the mean, ∑_*l*_ represents the covariance, *π*_*l*_ represents the mixing coefficient, and *π*_*l*_ satisfies ∑_*l*=1_^*L*^*π*_*l*_=1, 0 ≤ *π*_*l*_ ≤ 1.

From the viewpoint of sum and product, edge density can be expressed as follows:

(2) $$\begin{array}{c} \rho \left(x\right)=\sum_{l=1}^{L}p\left(l\right)p\left(\frac{x}{l}\right). \end{array}$$

*π*_*l*_=*p*(*l*) represents the prior probability that a data sample produces the *l* Gaussian component and *R*(*x*/*μ*_*l*_, ∑_*l*_)=*p*(*x*/*l*) represents the *x* probability under a given *l* rule. For a given GMM, it is also necessary to determine the unknowns contained in each Gaussian component of the model, such as mean, covariance, and mixing coefficient. This study uses the expectation-maximization algorithm based on maximum likelihood estimation to estimate the model parameters [16]. It is assumed that the sample set is *X*={*x*_1_, *x*_2_, *x*_3_, ⋯, *x*_*m*_} and the value set of the implied variable *r* is *R*={*r*_1_, *r*_2_, *r*_3_, ⋯, *r*_*m*_}, and then, the logarithmic likelihood function of the sample set can be expressed as follows:

(3) $$\begin{array}{c} K\left(\alpha \right)=\sum_{R}p\left(X,\frac{R}{\alpha }\right). \end{array}$$

*p*(*X*, *R*/*α*) represents a probability model with an unknown parameter *α* as the parameter, and *α*={*μ*_*l*_, ∑_*l*_, *π*_*l*_/*l*=1,2, ⋯, *L*}.

By maximizing the logarithmic likelihood function, the maximum likelihood solution of the parameter can be obtained. The iterative process of the expectation-maximization algorithm is mainly divided into two steps. The first step is to calculate the expectation of the likelihood function according to the initial value of the parameter or the last iteration value. The second step is to maximize the likelihood function to obtain new parameter values.

When the sample size is *M*, the expectation obtained through the first step can be expressed as follows:

(4) $$\begin{array}{c} P\left(\frac{\alpha }{\alpha^{m}}\right)=\sum_{l=1}^{L}\sum_{i=1}^{m}p\left(\frac{l}{x_{i},}\alpha^{m}\right)\log\;\pi_{i}+\sum_{l=1}^{L}\sum_{i=1}^{m}p\left(\frac{l}{x_{i},}\alpha^{m}\right)\log\;p_{l}\left(\frac{x_{i}}{\alpha_{l}}\right). \end{array}$$

*ρ*_*l*_(*x*_*i*_/*α*_*l*_) corresponds to the *l* Gaussian component and *ρ*(*l*/*x*_*i*_, *α*^*m*^) represents the posterior probability of the *l* Gaussian component.

(5) $$\begin{array}{c} p\left(l/x_{i},\alpha^{m}\right)=\frac{\pi_{l}p_{l}\left(x_{i}/\alpha_{l}\right)}{\sum_{l=1}^{L}\pi_{l}p_{l}\left(x_{i}/\alpha_{l}\right)}. \end{array}$$

After obtaining the expectation, the second step is used to calculate the model parameters.

(6) $$\begin{array}{c} \pi_{l}^{m+1}=\frac{\sum_{i=1}^{M}p_{l}\left(l/x_{i},\alpha_{l}^{m}\right)}{M},\\ \mu_{l}^{m+1}=\frac{\sum_{i=1}^{M}x_{i}p_{l}\left(l/x_{i},\alpha_{l}^{m}\right)}{\sum_{i=1}^{M}p_{l}\left(x_{i}/x_{i},\alpha_{l}^{m}\right)},\\ \sum_{l}^{m+1}=\frac{\sum_{i=1}^{M}{\left(x_{i}-\mu_{l}^{m+1}\right)}^{2}p_{l}\left(l/x_{i},\alpha_{l}^{m}\right)}{\sum_{i=1}^{M}p_{l}\left(l/x_{i}|,|\alpha_{l}^{m}\right)}. \end{array}$$

**Figure 2:**
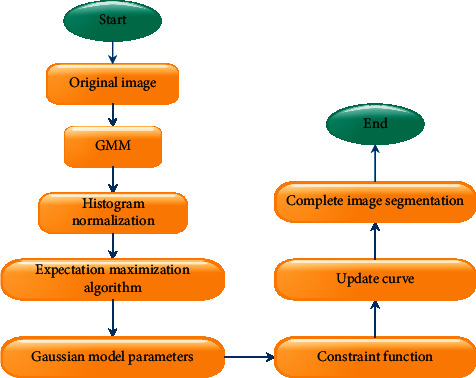
Nonparametric variable model image segmentation process based on histogram analysis.

The first step and the second step are iterated until the model parameters converge. Therefore, the nonparametric variable model image segmentation process based on histogram analysis is shown in Figure 2.

### 2.4. Algorithm Performance Indicators

The segmentation performance of the algorithm was assessed regarding sensitivity, specificity, and overall performance. Parametric deformation (PD) [17] and geometric deformation (GD) models [18] were compared with the proposed algorithm:

(7) $$\begin{array}{c} \text{sensitivity}=\frac{\text{TP}}{\text{TP}+\text{FN}},\\ \text{specificity}=\frac{\text{TN}}{\text{FP}+\text{TN}},\\ \text{overall performance}=\frac{\text{TP}+\text{TN}}{\text{FN}+\text{FP}+\text{TP}+\text{TN}}, \end{array}$$

where TP is true positive, TN is true negative, FP is false positive, and FN is false negative.

### 2.5. Clinical Data Collection

The age, gender, time interval from the onset of the first symptom to the follow-up MRI examination, past history (heart disease, stroke, transient ischemic attack, atherosclerosis, and other related diseases), National Institutes of Health Stroke Scale (NIHSS) score, and modified Rankin scale (mRS) score were collected.

### 2.6. Statistical Method

The data in this study were analyzed by SPSS 19.0. The measurement data were expressed as mean ± standard deviation ($\bar{x}\pm s$), and the count data were expressed as percentage. One-way analysis of variance was used for pairwise comparison. ROC curves were used to analyze the diagnostic effects of DWI, SWI, 3D TOF MRA, and MR SCALE-PWI sequences on ischemic penumbra in patients. The difference was statistically significant with *P* < 0.05.

## 3. Results

### 3.1. Basic Data of Patients

**Figure 3:**
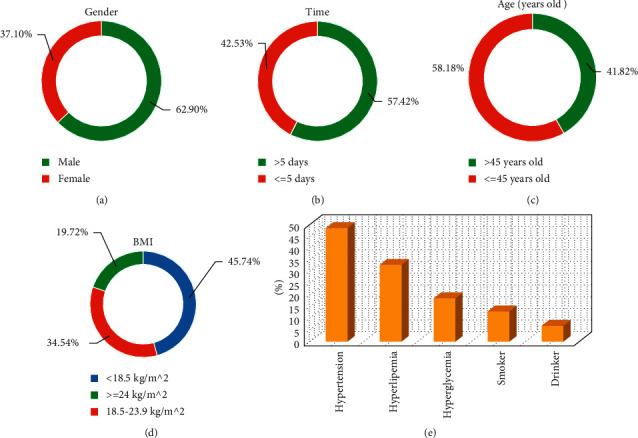
Basic information of patients. (a) Gender ratio; (b) the time interval from the onset of the first symptom to the follow-up MRI examination; (c) age; (d) BMI; (e) past medical history.

Figure 3 shows that the proportion of male patients (62.9%) was higher than that of female patients (37.1%). The proportion of patients with the first symptom onset to follow-up MRI examination interval greater than 5 days (57.42%) was higher than that of patients within 5 days (42.53%). The proportion of patients older than 45 years (58.18%) was higher than that of patients younger than 45 years (41.82%). The proportion of patients with BMI less than 18.5 kg/m^2^ (45.74%) was the highest, followed by patients with BMI 18.5–23.9 kg/m^2^ (42.53%), and the proportion of patients with BMI greater than 24 kg/m^2^ (19.72%) was the lowest.

According to the past medical history of the 116 patients, 56 patients had hypertension, accounting for 48.51%. There were 38 patients with hyperlipidemia, accounting for 32.86%. There were 22 patients with hyperglycemia, accounting for 18.62%. There were 15 smokers, accounting for 13.09%. There were 8 drinkers, accounting for 6.96%.

### 3.2. MRI Images of Patients

**Figure 4:**
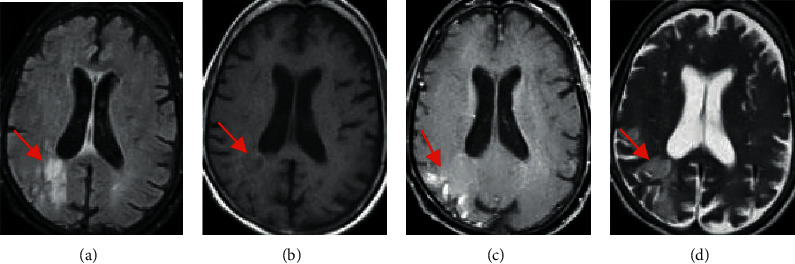
MRI image of a 58-year-old female patient. The arrows in the images indicate the relevant stroke area.

Figure 4 shows MRI images of a 58-year-old female patient with early subacute ischemic stroke. FLAIR in image A shows hyperintensity in the corresponding area, T1W1 in image B shows hypointensity in the corresponding area, T1 enhanced scan in image C shows parenchymal enhancement in the affected area, and T2W1 in image D shows hyperintensity in the corresponding area.

### 3.3. Algorithm Performance Analysis

**Figure 5:**
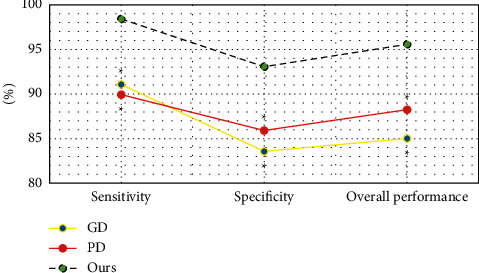
Diagnostic sensitivity, specificity, and overall performance of the algorithm.  ^*∗*^Compared with the algorithm, *P* < 0.05.

Figure 5 shows the comparison of the diagnostic sensitivity, specificity, and overall performance of the algorithm. The diagnostic sensitivity of the algorithm in this study was 98.41%, the specificity was 93.06%, and the overall performance was 95.58%. The diagnostic sensitivity of the GD algorithm was 91.07%, the specificity was 83.57%, and the overall performance was 85.02%. The diagnostic sensitivity, specificity, and overall performance of the PD algorithm were 89.93%, 85.91%, and 88.25%, respectively. The diagnostic sensitivity, specificity, and overall performance of the proposed algorithm were significantly higher than those of the GD algorithm and PD algorithm, and the difference was statistically significant (*P* < 0.05).

**Figure 6:**
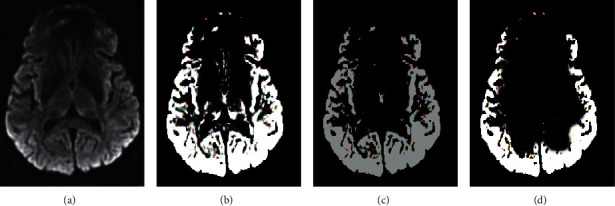
Segmentation effect of MRI images by the algorithm. (a) The original image; (b) the algorithm result; (c) the segmentation result of the GD algorithm; (d) the segmentation result of the PD algorithm.

Figure 6 shows the segmentation effect of the algorithm on MRI images. The segmentation results of the algorithm on MRI images were relatively complete, the display clarity of the lesion was better than other algorithms, and the overall quality was the best.

### 3.4. ROC Curve Analysis

**Figure 7:**
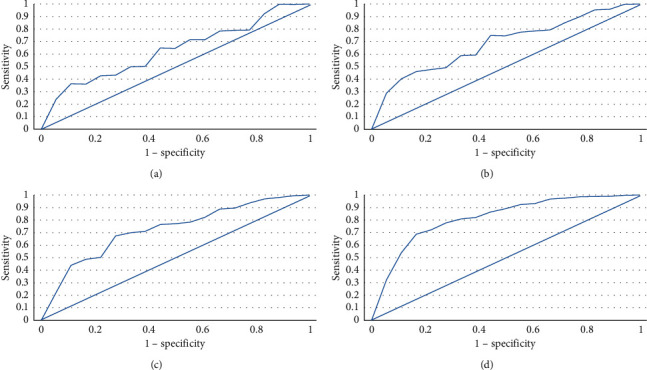
ROC curves of DWI, SWI, 3D TOF MRA, and MR SCALE-PWI sequences in the diagnosis of ischemic penumbra in patients. (a) DWI sequence; (b) SWI sequence; (c) 3D TOF MRA sequence; (d) MR SCALE-PWI sequence.

Figure 7 shows the ROC curve analysis of DWI, SWI, 3D TOF MRA, and MR SCALE-PWI sequences in the diagnosis of ischemic penumbra in patients. The AUC of the DWI sequence in the diagnosis of ischemic penumbra was 0.793, that of SWI was 0.825, that of 3D TOF MRA was 0.871, that of MR SCALE-PWI was 0.933, and that of MR SCALE-PWI was the highest.

## 4. Discussion

Cerebral ischemia is caused by the decrease of local or diffuse cerebral blood flow. When the blood flow is lower than a certain value, ischemia becomes irreversible infarction. Ischemic stroke refers to an acute neurological dysfunction caused by a single or multiple focal cerebral infarction [19]. At present, neuroimaging examination is a common clinical method for the diagnosis of ischemic stroke. Early use of CT, CT angiography, conventional MRI, DWI, and other means is helpful to prevent the occurrence and development of stroke, and MRI multimodal imaging is the most widely used [20]. The original image often has the problems of more noise, more artifacts, and low quality. It is necessary to introduce a mathematical model for enhancement processing. A nonparametric variable model based on histogram analysis is proposed, and its performance is compared with the PD and GD models. The diagnostic sensitivity, specificity, and overall performance of the proposed algorithm are significantly higher than those of the GD and PD algorithms, and the difference is statistically significant (*P* < 0.05), which is similar to the results obtained by Mathukumalli et al. [21]. It shows that the nonparametric variable model based on the histogram analysis designed in this study has a good segmentation effect on MRI images and can effectively improve the quality of the original image. By comparing the segmentation results of the algorithm for MRI images, the segmentation results of the algorithm in this study for MRI images are relatively complete, and the clarity of the lesion is better than that of other algorithms. The overall quality is the best, which is consistent with the results of quantitative data [22].

116 patients with acute ischemic stroke were selected as the subjects. Routine MRI (T2WI, T1WI, FLAIR, DWI, SWI, and 3D TOF MRA) and MR SCALE-PWI were performed. The data of patients were analyzed, and it was found that the proportion of male patients (62.9%) was higher than that of female patients (37.1%), which may be due to the large number of male smokers [23]. The proportion of patients older than 45 years(58.18%) was higher than that of patients younger than 45 years (41.82%), indicating that the incidence of ischemic stroke in the elderly population was higher. Among 116 patients, 56 patients were with hypertension, 38 patients with hyperlipidemia, 22 patients with hyperglycemia, 15 cases of smokers, and 8 cases of drinkers. The medical history was correlated with the occurrence of ischemic stroke [24]. ROC curve analysis showed that the AUC area of the DWI sequence in the diagnosis of ischemic penumbra was 0.793. The AUC area of the SWI sequence in the diagnosis of ischemic penumbra was 0.825. The AUC area of the 3D TOF MRA sequence in the diagnosis of ischemic penumbra was 0.871. The AUC area of the MR SCALE-PWI sequence in the diagnosis of ischemic penumbra was 0.933. This indicates that MR SCALE-PWI has a good effect on the early identification of ischemic penumbra, which can reduce unnecessary treatment and prolong the time window for patients to receive recanalization or neuroprotective treatment [25].

## 5. Conclusion

In this work, the nonparametric variable model based on histogram analysis was compared with PD and GD models, and 116 patients with acute ischemic stroke were given routine MRI scans at the same time. The results showed that the variable model algorithm had a strong ability to completely segment MRI images and display lesions and can effectively identify the occurrence of ischemic stroke. Moreover, the MR SCALE-PWI sequence had a better early identification effect on the ischemic penumbra, regarding which an effective treatment plan can be formulated. However, the disadvantage is that the sample size of patients is small, and the results may be biased. The PET data of patients are not collected, and the evaluation value of the gold standard and MRI cannot be compared. Third, the MRI equipment used is the latest purchase, and the LOVARS MRI data are not collected. In the future, the sample size of patients will be expanded, and the identification performance of the MR SCALE-PWI sequence in ischemic core and ischemic penumbra will be further explored. In conclusion, this study provides a reference for the clinical diagnosis of ischemic stroke patients.

## Figures and Tables

**Table 1:** Conventional MRI parameters.

Parameter	Axis position T2WI	Axis position T1WI	Axis position FLAIR	Sagittal position T1WI	Axis position DWI	SWI	3D TOF MRA
TR (ms)	4500	400	8400	200	2400	25	21
TE (ms)	97	2.5	105	2.48	50	18.5	3.5
FOV (mm)	250 × 250	250 × 250	250 × 250	250 × 250	250 × 250	250 × 250	220 × 220
Layer thickness (mm)	6	6	6	6	6	2	0.5
Number of incentives	1	1	1	1	1	1	1
Matrix	320 × 320	320 × 320	320 × 320	320 × 320	160 × 160	521 × 521	320 × 320
Scanning time (s)	62	48	115	50	64	216	216

**Table 2:** MR SCALE-PWI scan parameters.

Parameter	MR SCALE-PWI
TR (ms)	1500
TE (ms)	32
FOV (mm)	230 × 230
Layer thickness (mm)	6
Number of incentives	1
Matrix	125 × 125
Scanning time (s)	135

## Data Availability

The data used to support the findings of this study are available from the corresponding author upon request.
